# Executive Control of Actions Across Time and Space

**DOI:** 10.1177/0963721416659254

**Published:** 2016-12-05

**Authors:** Frederick Verbruggen

**Affiliations:** Department of Psychology, College of Life and Environmental Sciences, University of Exeter, and Department of Experimental Psychology, Ghent University, Ghent, Belgium

**Keywords:** executive control, impulsive action, learning, biocultural evolution, sociocultural influences

## Abstract

Many popular psychological accounts attribute adaptive human behavior to an “executive-control” system that regulates a lower-level “impulsive” or “associative” system. However, recent findings argue against this strictly hierarchical view. Instead, executive control of impulsive and inappropriate actions depends on an interplay between multiple basic cognitive processes. The outcome of these processes can be biased in advance. Executive-action control is also strongly influenced by personal experiences in the recent and distant past. Thus, executive control emerges from an interactive and competitive network. Main challenges for future research are to describe and understand these interactions and to put executive-action control in a wider sociocultural and evolutional context.

Scientists and non-scientists alike have always shown great interest in how the human mind regulates behavior (Verbruggen, McLaren, & Chambers, 2014). This interest goes back to the ancient philosophers and writers, including Confucius, Plato, and Ovid. Nowadays, psychologists typically attribute adaptive behavior to an executive system, which can override impulsive or inappropriate actions, allowing people to fulfill long-term goals. In contemporary Western societies, executive control (or a lack of it) has been linked to physical and mental health outcomes, school and job success, substance (ab)use, and personal finances (Moffitt et al., 2011). Furthermore, the idea that people have voluntary control over their impulses and actions permeates our current social systems (Logan, 2003). Thus, executive control is critical in everyday life. Nevertheless, this aspect of human functioning has proven to be one of the most difficult issues to understand. In this article, I will provide a selective review of research on executive control of actions (for in-depth discussions and detailed overviews, see the Recommended Reading list). I will primarily focus on the stopping of inappropriate or impulsive actions (i.e., *response inhibition*). Stopping is generally considered a simple but extreme act of executive control, and has proven to be an excellent case study.

## Mechanisms of Executive Action Control

Historically, control of impulsive or inappropriate actions was attributed to an intentional and rational executive-control system that regulated a lower-level automatic and emotionally charged system. However, recent findings have suggested that this strictly hierarchical view is incorrect. Instead, action control seems to emerge from a highly interactive network with no clear boundaries between “higher-level” and “lower-level” systems. Nevertheless, the traditional dual-systems accounts still prevail in the wider literature.

### How to control impulsive or inappropriate actions

In the past two decades, great efforts have been made to fractionate the executive controller into distinct control functions (e.g., Miyake et al., 2000). But too often researchers label operations as “executive” without further questioning the nature of the underlying processes. Consequently, they often fail to explain *how* behavior is regulated in complex environments. Progress on the control problem requires a more precise approach.

Researchers should focus on the building blocks of action control. It is generally accepted that acting or responding to a stimulus involves different processing stages (e.g., Sternberg, 1969). Similarly, action control in response to changes in the environment or internal state involves a chain of basic processes that results in the inhibition of a response and the activation of an alternative one (Verbruggen, McLaren, & Chambers, 2014). For example, the first step of various forms of action control, such as stopping, involves detecting relevant information that signals the need for control; these signals can be external (e.g., a red traffic light) or internal (e.g., a sudden thought or conflict between various response options). Next, an appropriate action (e.g., stopping) needs to be selected or retrieved from memory. Finally, the selected action (e.g., pressing the brake pedal) has to be executed quickly but accurately.

These basic control processes are inherently competitive and interactive. The competition idea has received support from behavioral, neuroscience, and computational studies. For example, when neural activity associated with one visual stimulus increases, activity associated with other concurrent stimuli decreases (Duncan, 2006). This could also explain why people find it difficult to attend to more than one stimulus or do more than one thing at the same time. The interactive idea has received support from recent studies, including from my own lab. A prominent model of response inhibition and executive control, the independent-race model, assumes that executive-control processes (stop) and lower-level processes (go) are independent (Logan & Cowan, 1984). However, we found strong dependence between go and stop processes when task difficulty was manipulated (Verbruggen & Logan, 2015). Other studies have also observed brief moments of interaction in easier response-inhibition tasks (e.g., Boucher, Palmeri, Logan, & Schall, 2007). Thus, action control seems to emerge from a competitive and interactive network, rather than from an independent top-down control system that oversees and alters ongoing processing in lower-level systems.

### Bias and anticipatory control

Often people must find a delicate balance between competing task demands. Focusing on a single stimulus could lead to overly rigid behavior, whereas the constant reorienting of attention could lead to constant distraction. Similarly, responding quickly in the currently relevant task (e.g., driving home) can lead to fast task completion, but it reduces the likelihood that an action can be stopped or replaced in response to unexpected changes in the environment (e.g., a child crossing the street).

Such a balance can be achieved by biasing stimulus or response competition in advance. For example, when the organism predicts the occurrence of a stimulus (e.g., based on previous experiences or external cues, such as a traffic warning sign), it can pre-activate the relevant visual cells, biasing neural competition in favor of the expected stimulus (e.g., Duncan, 2006). Similarly, when the system predicts certain actions, it can pre-activate the motor network, biasing action selection and reducing the response latency of the anticipated action (e.g., Bestmann, 2012).

The biasing idea can account for a range of phenomena in the control literature. For example, we proposed a general biasing account for proactive inhibitory control (Elchlepp, Lavric, Chambers, & Verbruggen, 2016). When subjects are informed that they may have to stop a response in the near future, they typically slow down (Aron, 2011; Verbruggen & Logan, 2009b). Our results indicated that proactive inhibitory control works by biasing or altering (neural) activity in lower-level systems that are involved in stimulus detection, action selection, and action execution. For example, subjects monitored for perceptual features of the stop signal in stop contexts. They also traded speed in the go task for success in the stop task. These findings are consistent with work in the wider attention and executive-control literature (see Elchlepp et al., 2016, for a discussion). Furthermore, we found that subjects made similar proactive-control adjustments in a task in which they occasionally had to execute an additional response (instead of stopping a response). Finally, we observed an overlap between proactive inhibitory control and proactive control in task-switching studies (i.e., preparation for upcoming tasks; for reviews on task switching, see Kiesel et al., 2010; Vandierendonck, Liefooghe, & Verbruggen, 2010). These findings led us to conclude that all forms of proactive control require reconfiguration or biasing of task settings (e.g., which stimulus to attend to, which response to execute, etc.). Thus, the most important difference between tasks or contexts is which processing systems are adjusted, rather than which adjustment mechanisms are involved (Elchlepp et al., 2016; for a similar argument, see Logan, Van Zandt, Verbruggen, & Wagenmakers, 2014).

When attention is proactively allocated and responses are prepared, behavior may not require much control anymore; instead, responses could be activated easily by stimuli in the environment (Meiran, Cole, & Braver, 2012). Indeed, response inhibition can be triggered by task-irrelevant primes (e.g., van Gaal, Ridderinkhof, van den Wildenberg, & Lamme, 2009; Verbruggen & Logan, 2009a), but these priming effects are primarily observed in contexts in which subjects are instructed to stop occasionally (Chiu & Aron, 2014; Verbruggen & Logan, 2009a). These findings are consistent with the *prepared-reflex* idea: Once subjects have made proactive-control adjustments in anticipation of a stop signal, the stop response can be activated easily by both task-relevant and task-irrelevant information in the environment.

### Influences of the recent past

Performance monitoring is a critical component of action control (Ullsperger, Danielmeier, & Jocham, 2014). For example, people often slow down after they make an error. This slowing is usually attributed to the executive system: When it detects an error or suboptimal outcome, it adjusts the parameters of perceptual and response systems to reduce the likelihood of future errors. Thus, errors or suboptimal outcomes can signal the need for extra control. Consistent with this idea, subjects often slow down after an unsuccessful stop. However, slowing has been observed after successful stopping as well. This led Bissett and Logan (2012) to conclude that stop-signal presentation encourages subjects to shift priority from the go task to the stop task (i.e., it biases competition). Such a shift produces longer response latencies after a signal trial and can reduce the latency of the stop process.

Recent events can influence actions in other ways as well. When people perform an action, they store information about the stimulus, the task, the selected action, and possibly the control settings in memory (Egner, 2014; Logan, 1988). When the stimulus is repeated, this information is retrieved. This could explain why people are often slower to respond to a stimulus that was previously presented in a different task context or that was previously paired with another response or with stopping.

Another source of sequential effects is residual activation in perceptual or motor systems. For example, we found that excitability of the motor system was associated with the response properties of the previous experimental trial (Verbruggen, McAndrew, Weidemann, Stevens, & McLaren, 2016). In competitive systems, small activation differences at the beginning of a trial could make the difference between whether or not a response is selected. The same study showed that previous events influenced actions even if this went against conscious expectancies about upcoming events. Proactively biasing response options is effortful (Braver, 2012). Thus, even though people can predict what will happen next, they may not always adjust their behavior accordingly. This could explain why recent events can influence our behavior, even when this is inappropriate.

### Learning and development at the heart of executive control

A main executive-control function is biasing activity based on rules, expectancies, or task goals. But how do people know which options or processing pathways to bias? Some progress has been made in answering this question. For example, Rougier, Noelle, Braver, Cohen, and O’Reilly (2005) developed a neurologically inspired model of rule learning and control. The model was trained to respond to multidimensional stimuli. In each block, only one dimension was relevant (e.g., color), but the specific features within the dimension could change (e.g., red, green, yellow). Throughout the block, the prefrontal cortex system developed patterns of activity that encoded abstract representations of the relevant stimulus dimension (e.g., “color”). These abstract rule-like representations subsequently guided behavior by providing excitatory support for the relevant dimension in the stimulus-processing layers; in other words, they biased competition. This was possible because links between the abstract representations and the processing layers were built during training (note that a similar principle could explain how stimuli, cues, or task contexts can become associated with control settings, as described above).

Other work has indicated that acquired representations (and the links between the representations and processing layers) can be reused with a variety of other representations; furthermore, they can be reapplied in different contexts (Cole, Laurent, & Stocco, 2013). This could explain people’s ability to rapidly learn new action rules from instructions and show adaptive behavior in novel situations.

But learning also influences performance in a stimulus-specific way. Logan (1990) suggested that the stimulus-response bindings (as described above) could be the first step toward automatization. Consistent with this idea, our work suggests that executive control of impulsive and inappropriate actions can become progressively more “automatic” throughout practice (Verbruggen & Logan, 2008). In a series of studies, we have demonstrated that people learn to associate specific stimuli with different aspects of stopping (for a discussion of what is learned, see Verbruggen, Best, Bowditch, Stevens, & McLaren, 2014). When the stimulus is repeated, these associations are retrieved, and the stop network is activated automatically. This work is theoretically relevant because it shows that “executive” functions, such as response inhibition, can become automatized. It also has practical implications. Recent meta-analyses have indicated that stimulus-specific stop training can influence food and alcohol consumption (e.g., Jones et al., 2016). Thus, outsourcing control to the environment may help people regulate their actions.

Integrating the learning and control literatures may also provide new insights into the development of executive control. The ability of children to control their actions improves remarkably from infancy through adolescence to adulthood. Several key transitions have been identified, such as from reactive to proactive control,^1^ from externally driven to self-directed control, and from stimulus-driven to rule-based behavior (Munakata, Snyder, & Chatham, 2012). The work discussed above indicates how children can go from implementing specific stimulus-response associations to general rules: Children may gradually develop abstract representations through constant interaction with their environment and associate these with relevant behavior (i.e., they may learn that similar objects or situations require a similar response). Such abstract representations can subsequently guide or contextualize stimulus detection, action selection, and action execution.

### Interim conclusions

The work reviewed above indicates that control of impulsive or inappropriate actions involves a chain of processes that occur on different time scales. Researchers should explore at which of the processing stages situational, individual, or group differences arise. Furthermore, there is no clear distinction between “controlled” (or “goal-directed”) and “automatic” processes. For example, automatic processes can be goal-directed and are influenced by task context (Ridderinkhof, 2014), whereas the prepared-reflex and associated-learning work has shown that some “control” processes (e.g., stopping) can operate in a (semi-)automatic way. Finally, other research not reviewed here has suggested that emotion and motivational processes can have a strong influence on executive control (e.g., Braver et al., 2014). Combined, these findings indicate that control of impulsive actions arises from a highly interactive network with multiple components and influences, rather than from two separate systems that operate in parallel.

## Future Directions and Challenges

A main challenge for future research is to describe how executive control emerges from an interactive and competitive network. Furthermore, it remains unclear how individuals build up a control repertoire throughout development and what is learned when people control their actions. Finally, models of control assume that processing is biased based on rules or (task) goals. But how are these rules or goals selected? In the case of externally driven control, the answer is usually straightforward (e.g., a parent giving instructions). For internally driven control, the answer is less obvious, although some progress has been made in tackling this issue (Ridderinkhof, 2014). Ultimately, progress on the control problem will require addressing fundamental questions about the nature of volition and intentionality.

Significant progress in understanding executive control will also require new models that integrate different disciplines and literatures. Behavior is controlled not just by the “self” but also by society. First, society dictates what should be controlled. Children’s behavior is initially regulated via *external controllers* (e.g., parents or teachers). By the time they reach adulthood, most people have learned what behavior is acceptable and what should be inhibited (e.g., taboos^2^ or drug consumption). Second, society can influence how urges and impulses are controlled. Proactive forms of control (i.e., regulating the environment or behavior before the actual impulse or urge arises) are likely to be significant determinants of impulsive and problematic behaviors (e.g., substance misuse) in the real world. Society can fulfil such a proactive role by regulating the environment (e.g., by enforcing plain cigarette packaging). Third, society determines to a certain degree how we define (perceived) impulse-control problems. For example, certain forms of impulsive behavior (e.g., attention-deficit/hyperactivity disorder and substance misuse) are now understood in some societies as neurological disorders, best treated with medications. This raises fundamental questions about volition and responsibility (disease vs. lifestyle choice). Importantly, beliefs about agency, self-control, and “free will” can have a strong impact on control processes and behavior itself (Rigoni, Braem, Pourtois, & Brass, 2016).

Executive control is also shaped by genetic evolution. Different species (e.g., birds, lemurs, and monkeys) can perform tasks in which they have to suppress inappropriate actions, indicating that executive control has evolved across species (MacLean et al., 2014). This finding undermines the traditional belief that executive control is a uniquely human trait that distinguishes us from “impulsive” animals. However, we don’t have a coherent account of the bio-evolution of executive control yet. In addition to genetic evolution, a second mechanism of inheritance, culture, gives rise to social norms, rules, and laws that have developed and changed throughout history. Therefore, we also have to study differences across time, space, and species and explore the origin and consequences of such variations.

## Conclusion

I propose that executive control of actions arises from a chain of interactive and competitive processes. Furthermore, I argue that executive control is strongly influenced by events in the past, by the broader context in which the individual operates, and by biocultural evolution. Even though most researchers will agree that mental functions such as executive control are influenced by the environment and by evolution, the various external and internal influences are rarely discussed together. Only when we understand the interaction between individuals and their environment can we have a really satisfactory theory of executive control.

## Notes

1. Some have argued that when people age, control shifts again from proactive control to reactive control; this shift may account for age-related differences between young and older adults in a variety of executive-control tasks (Braver, 2012).

2. Neuroimaging findings suggest that taboo words activate parts of the neural network that is involved in canceling actions in response to external signals (Severens, Kühn, Hartsuiker, & Brass, 2011)
